# Biophysical Properties of *Lumbricus terrestris* Erythrocruorin and Its Potential Use as a Red Blood Cell Substitute

**DOI:** 10.3390/jfb3010049

**Published:** 2012-01-06

**Authors:** Jacob Elmer, Andre F. Palmer

**Affiliations:** William G. Lowrie Department of Chemical and Biomolecular Engineering, The Ohio State University, 425 Koffolt Laboratories, 140 West 19th Avenue, Columbus, OH 43210, USA; E-Mail: elmer.18@osu.edu

**Keywords:** red blood cell substitute, hemoglobin, erythrocruorin, oxygen carrier

## Abstract

Previous generations of hemoglobin (Hb)-based oxygen carriers (HBOCs) have been plagued by key biophysical limitations that result in severe side-effects once transfused *in vivo*, including protein instability, high heme oxidation rates, and nitric oxide (NO) scavenging. All of these problems emerge after mammalian Hbs are removed from red blood cells (RBCs) and used for HBOC synthesis/formulation. Therefore, extracellular Hbs (erythrocruorins) from organisms which lack RBCs might serve as better HBOCs. This review focuses on the erythrocruorin of *Lumbricus terrestris* (LtEc), which has been shown to be extremely stable, resistant to oxidation, and may interact with NO differently than mammalian Hbs. All of these beneficial properties show that LtEc is a promising new HBOC which warrants further investigation.

## 1. Extracellular Hemoglobins: A New Paradigm

As of 2011, the only hemoglobin (Hb) based oxygen carriers (HBOCs) that have entered phase III clinical trials are polymerized human [1–4] and bovine [5–8] Hb (PolyHb) as well as poly(ethylene glycol) surface-conjugated human hemoglobin (MP4, Sangart Inc., San Diego, CA, USA) [9–12]. MP4 is currently undergoing clinical trials, but the PolyHbs have been discontinued due to indications of increased mortality and other complications [13]. The major problems associated with these HBOCs (instability, oxidative stress, and nitric oxide (NO) scavenging) can be directly attributed to removing Hb from the protective environment within the red blood cell (RBC). The RBC has enzymes to prevent oxidation [14–16], a cell membrane to reduce interactions with NO [17], allosteric effectors to modulate O_2_ delivery [18], and high Hb concentrations that minimize dimerization of the Hb tetramer [19]. 

Since mammalian Hbs purified from RBCs are burdened with so many problems, extracellular Hbs from other organisms may be better suited for use in HBOC development. This special class of Hbs, known as erythrocruorins (Ecs), are found in organisms which lack RBCs (most annelids [20], some mollusks [21] and insects [22]). Consequently, Ecs have already adapted to the harsh conditions in the bloodstream with unique structural and functional modifications that make them attractive natural HBOCs. This review will focus on the unique properties of Ec from the Earthworm *Lumbricus terrestris* (LtEc).

## 2. Structure and Stability of LtEc

Ecs come in a wide variety of shapes and sizes, including the spherical Ec of *Riftia pachyptila* (~400 kDa) [23], the hexagonal bilayer (HBL) Ecs of *L. terrestris* [24] or *Arenicola marina* [25], and the huge cylindrical Ec of the clam *Cardita borealis* (12 MDa) [26]. These Ecs are all held together by covalent disulfide bonds and strong electrostatic or hydrophobic forces within large subunit interfaces. Therefore, they are not susceptible to dissociation at low concentrations like mammalian Hbs, which lack intermolecular disulfide bonds [27].

**Figure 1:**
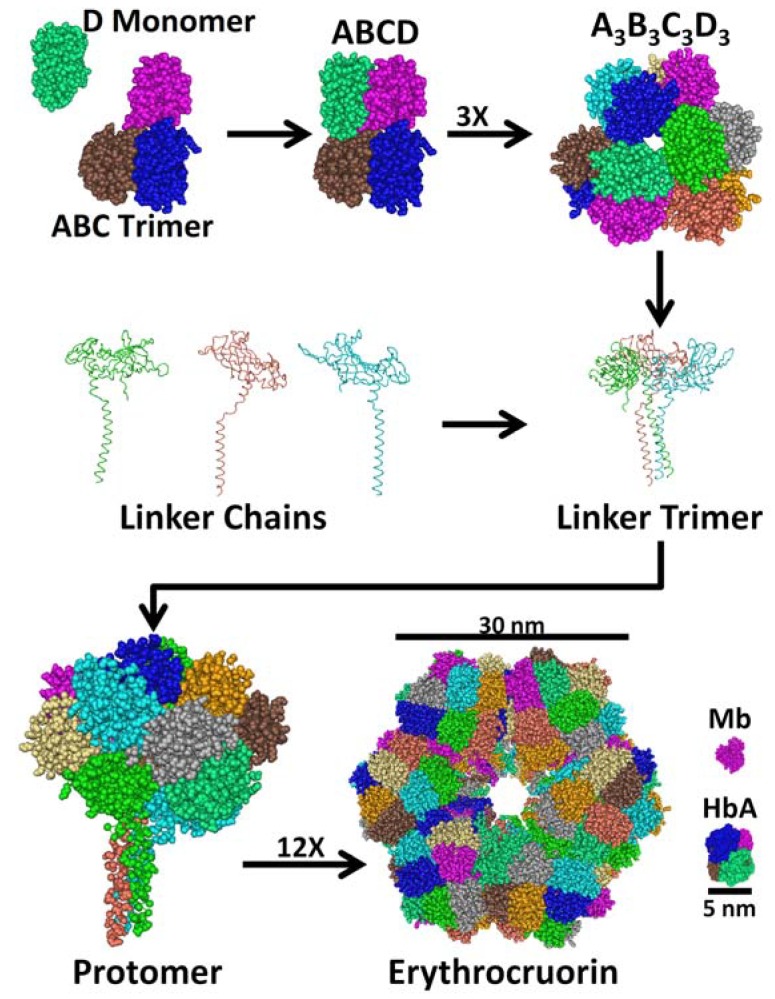
Assembly of *Lumbricus terrestris* erythrocruorin (LtEc). LtEc consists of 5 globin subunits (A, B, C, D_1_’, and D_2_) and 4 linker chains (L_1_, L_2_, L_3_, or L_4_). The subunits self assemble into an ABC trimer that pairs with a D monomer to form the ABCD tetramer which then associates with two more tetramers to form the dodecamer. Three linker subunits form a linker trimer which binds the dodecamer to form a protomer. Finally, 12 protomers assemble into the hexagonal bilayer structure of LtEc, which has a MW of 3.6 MDa and a diameter of approximately 30 nm [24]. HbA [27] and myoglobin (Mb) [38] are shown to the right to provide a sense of scale.

LtEc consists of a macromolecular assembly of 144 globin subunits and 36 linker chains (Figure 1) [24,28,29]. There are 5 types of globins (A, B, C, and D_1_ or D_2_) [30,31] and 4 types of linkers (L1, L2, L3, and L4) [32,33]. Each globin subunit has a single intramolecular disulfide bond and a structure that is more similar to myoglobin than mammalian Hb subunits [28]. Each subunit also contains a heme group, which binds oxygen (O_2_) and even contributes to subunit association by forming hydrogen bonds with adjacent subunits through propionate groups [29]. The A, B, and C subunits also have intermolecular disulfide bonds which form an ABC trimer. The ABC trimer and D monomer self-associate through electrostatic and hydrophobic interactions to form the ABCD tetramer [34]. Next, the A_3_B_3_C_3_D_3_ dodecamer spontaneously forms from three ABCD tetramers through disulfide bonds. The dodecamer is hemi-spherical and has a structure that is reminiscent of the spherical (double dodecamer) Ec of *R. pachyptila* (RpEc) or *Oligobrachia mashikoi* (OmEc), suggesting that LtEc may have also been spherical at some point during its evolution [23,35,36].

The linker chains are not required for dodecamer formation [37], but they are necessary to form the complete hexagonal bilayer structure of LtEc. Initially, three linker chains self-assemble to form a linker trimer. The linker chains are degenerate, meaning that several combinations of L_1_, L_2_, L_3_ or L_4_ can create the trimer. In fact, the minimum requirement for linker trimer formation is only a binary mixture of L_1_ or L_2_ with L_3_ or L_4_ [37]. The purpose and origin of the degeneracy in the linker and globin subunits are not known and any possible effects of different subunit compositions will need to be considered in future studies. The linker trimer is held together by numerous disulfide bonds and strong hydrophobic interactions within a coiled coil domain [24]. The linker trimer also has large low density lipoprotein (LDL) domains which strongly bind the dodecamer to form the protomer. Finally, 12 protomers assemble through interactions between the coiled coil domains of the linker trimers to form the hexagonal bilayer structure of LtEc, which has a molecular weight (MW) of approximately 3.6 MDa and a diameter of 30 nm as shown in Table 1 [24]. To put these numbers into context, human Hb (HbA) has a MW of 0.064 MDa and a diameter of 5 nm [27]. 

**Table 1:** Size, molecular weight (MW), O_2_ affinity (P_50_), and cooperativity (calculated as the constant *n* from the Hill Equation) of HbA, AmEc, LtEc, and human RBCs.

	MW (kDa)	Diameter (nm)	P_50_ (mm Hg)	*n*(---)
HbA	64	5	11	2.7
AmEc	3,600 [39]	30 [39]	2.6	2.5 [39]
LtEc	3,600	30	28 [40]	3.7
RBC	---	8,000	26 [41]	2.75 [41]

Several other elements also contribute to the structure of LtEc. Approximately 50 calcium ions (Ca^2+^) are bound at various sites throughout LtEc. Copper and zinc atoms are also bound to LtEc [42]. The Ca^2+^ ions increase the stability of LtEc and help it resist unfolding at high temperatures [34,43]. Barium (Ba^2+^) has similar effects and addition of EDTA (which chelates divalent cations) decreases the thermal stability of LtEc [43]. LtEc is also extremely stable in the presence of chemical denaturants, exhibiting a half-life of 28 hours in 1.75 M urea [34]. However, LtEc is prone to subunit dissociation at alkaline pH (>8.0) [44]. In the oxidized (Fe^3+^) form, LtEc is also susceptible to higher rates of hemin release than oxidized HbA (LtEc = 20–40 × 10^−3^ min^−1^, HbA = 7.7 × 10^−3^ min^−1^) [45]. It is important to mention that some Ecs may be unstable *in vivo*. For example, the marine worm *A. marina* expresses an Ec (AmEc) which is adapted to a high ionic strength and quickly dissociates into dodecamers in human plasma, which has relatively low ionic strength [39]. In contrast, LtEc comes from the terrestrial Earthworm and is stable at the ionic strength of human blood [40].

## 3. O_2_ Transport by LtEc

Human blood and LtEc bind and release O_2_ in a similar fashion (see Table 1). The O_2_ affinity or P_50_ (pO_2_ at which half of the hemes are saturated with O_2_) of human blood (26 mm Hg) is almost identical to LtEc (28 mm Hg) [40]. This is in contrast to pure HbA and AmEc, which both have significantly lower P_50_ values (higher O_2_ affinities) than human blood. The O_2_ affinity of HbA decreases when it is purified from human blood due to the removal of its allosteric effector 2,3-DPG [18]. The allosteric effector of LtEc is Ca^2+^, which increases the O_2_ affinity of LtEc and is available in the bloodstream. Other divalent cations, like Ba^2+^, Sr^2+^, and Mg^2+^, have a similar effect on the O_2_ affinity of LtEc [46,47]. The relatively high O_2_ affinity (low P_50_) of AmEc is probably another effect of its exposure to low ionic strength buffers or an adaptation to the low O_2_ environment in which *A. marina* is found [39].

Cooperative oxygen binding is a unique trait of Hbs in which small changes in one subunit (*i.e.*, ligand binding) affect the conformations and ligand affinities of adjacent subunits. This phenomenon allows Hbs to become saturated with O_2_ in the lungs, hold onto it in the arteries, then release it in large amounts in the arterioles and capillaries. The cooperativities of HbA, AmEc, and blood are all around 2.5–2.7 under physiological conditions. The cooperativity of LtEc is relatively higher under physiological conditions (3.7), due to the increased number of subunit interactions within the LtEc dodecamer. In fact, the maximum cooperativity of LtEc is 7.9 at 25 °C and pH 7.7 with 25 mM CaCl_2_ [48]. The effects of cooperativity also appear to be mostly within the dodecamers and only slightly (if at all) transmitted between dodecamers [49]. 

As previously mentioned, the LtEc dodecamer spontaneously forms in the absence of the linker chains. Interestingly, isolated dodecamers and ABCD tetramers have O_2_ affinities and cooperativities similar to LtEc in its full form. The isolated ABC trimer and D monomer, however, have significantly higher O_2_ affinities and lower cooperativities. Therefore, the linker chains are not required for O_2_ transport and appear to simply increase the stability and size of LtEc [49].

## 4. Autoxidation of LtEc

Oxidation of the heme iron (Fe^2+^ → Fe^3+^) is an inevitable side-effect of O_2_ transport for all Hbs. After O_2_ binds to the heme iron, it can strip away an electron and escape the heme pocket, forming the pro-oxidant superoxide (O_2_^−^) and oxidized Hb (metHb, Fe^3+^). MetHb can be further oxidized to the ferryl form (Fe^4+^) and/or generate toxic hemichrome and other free radicals which greatly increase lipid oxidation in cell membranes and overall oxidative stress [50]. The size, structure, and amino acid composition of the heme pocket all have significant effects on the rate of Hb autoxidation. Large heme pockets allow O_2_^−^ to easily escape [51,52], while aromatic amino acids (*i.e.*, tyrosine or phenylalanine) within the heme pocket stabilize O_2_^−^ and reduce oxidation rates [52].

The heme pockets of LtEc are much smaller than HbA heme pockets [24,27]. Each subunit of LtEc also has phenylalanine or tryptophan residues which are not present in the heme pockets of HbA subunits [24]. These differences are clearly expressed in the redox potentials of HbA and LtEc (see Table 2). The redox potential of a species is a measure of how likely it is to accept or donate electrons. Species with positive redox potentials are more likely to accept electrons (reduction), while negative redox potentials indicate that a species is more likely to donate electrons (oxidation). The redox potential of HbA is negative (−50 mV), whereas LtEc has a highly positive redox potential (+112 mV). Therefore, LtEc is much less likely to undergo autoxidation than HbA [53–55]. In fact, experiments have shown that the autoxidation rate of LtEc (<0.010 h^−1^) is lower than HbA (0.014 h^−1^) [53]. LtEc may also be reduced by reducing agents that are found in the bloodstream (ascorbic acid or glutathione), while HbA is not as easily reduced [53,54].

**Table 2:** Autoxidation rates and redox potentials of HbA, LtEc, and AmEc.

	k_ox _(h^−1^)	E_o _(mV)
HbA	0.014 [39]	−50 [53]
LtEc	≤0.010	+112 [53]
AmEc	0.005 [56]	+63 [53]

Divalent cations also influence oxidation of LtEc. For example, Ba^2+^ and Ca^2+^ both reduce the rate of LtEc autoxidation. Sr^2+^ and Mg^2+^ have a similar, yet less significant effect [43]. The Cu and Zn atoms which are bound to LtEc also appear to have some superoxide dismutase (SOD) activity. SODs are a family of enzymes which react with O_2_^−^ to form water, thereby preventing formation of harmful H_2_O_2_ from O_2_^−^. The SOD activity of LtEc is approximately 10% of the human SOD enzyme, but any intrinsic anti-oxidant activity is beneficial from an HBOC development perspective [57].

## 5. Interactions Between LtEc and other Ligands

Hbs are known to bind, transport, and/or react with several other ligands besides O_2_. For example, Hbs bind both O_2_ and carbon monoxide (CO), but release CO much more slowly than O_2_. This competitive inhibition of O_2_ binding is the reason CO is a poisonous gas. LtEc can also bind CO and its subunits appear to have varying affinities (either high or low) for CO [58,59].

The interactions between Hb and NO have recently become crucially important with respect to HBOC development. Mammalian Hbs have been shown to catalyze a NO dioxygenation reaction in which O_2_ and NO react to form NO_3_^−^ and metHb. The metHb increases oxidative stress, but the elimination of NO can have much more significant effects *in vivo* [60]. NO is a signaling molecule which regulates the diameter of blood vessels, relaxing them at high concentrations (vasodilation) and constricting them at low concentrations (vasoconstriction). Vasoconstriction also increases blood pressure and causes harmful systemic hypertension. Therefore, reducing NO dioxygenation is a high priority in HBOC design.

Mutagenesis studies in HbA have shown that mutations that reduce autoxidation also reduce the rate of NO dioxygenation. For example, substituting large apolar or aromatic residues (leucine, tryptophan, or phenylalanine) within the heme pocket or charged amino acids (glutamine) near the heme pocket entrance greatly reduces the rate of NO dioxygenation in oxy-HbA [60,61]. Both LtEc and AmEc have naturally occurring phenylalanine and tryptophan residues within their heme pockets [24,25]. There have not yet been any studies which focus specifically on the interactions between Ec’s and NO, but there have been suggestions that Ec’s may have a significantly reduced rate of NO dioxygenation relative to HbA [24,39,62]. However, it is important to mention that these studies were not conclusive and more experiments must be done to determine the exact nature of the interactions between Ec’s and NO.

## 6. Availability and Economic Analysis of LtEc

A wide array of different Ec’s have been discovered with unique characteristics, such as extreme heat tolerance or H_2_S transport [36,63,64]. However, since a large amount of Ec will be needed to meet the global demand for a RBC substitute, the host organism must be readily available. Most of the Ec’s in the literature are extracted from deep sea worms, which may be difficult to grow at an industrial scale. Fortunately, LtEc is extracted from terrestrial earthworms which are readily available at a low cost (~$75/1,000 earthworms) from many commercial sources. It is important to mention, however, that many different species of terrestrial earthworms have been discovered which might be grown as easily as *L. terrestris*. For example, the Ec of the Brazillian earthworm *Glossoscolex paulistus* (GpEc) has been extensively studied [65–72] and is an interesting alternative to LtEc that may also be commercially viable.

Any Ec product must also be ultrapure to be considered a viable HBOC candidate. The purification strategy used for Ec production must be efficient to keep the costs of the Ec product comparable to a unit of donated blood (estimates vary from $200–$1,000) [73]. A few different Ec purification processes have been developed, including ultracentrifugation [49], size exclusion chromatography (SEC) [40], and tangential flow filtration (TFF) [62]. Ultracentrifugation is simple and quick, but requires expensive equipment and may not produce ultrapure material. SEC produces ultrapure Ec, however, it is limited by low yields and is difficult to scale up. In contrast, TFF can easily produce large amounts of ultrapure LtEc (5–10 g/1,000 worms) and is easily scalable. Using the TFF LtEc yield, assuming a unit of donated human blood contains approximately 40–80 grams of HbA, and omitting operational costs (which should be low for a TFF process), we can estimate that it would take at least 8,000 worms to produce a “unit” of LtEc with a cost of $600 [62]. A more thorough economic analysis would be needed before producing any Ec on an industrial scale, but this estimate suggests that LtEc may be a slightly more expensive, yet affordable, alternative to donated blood. 

## 7. Preliminary Animal Studies with Ec’s

Preliminary experiments have been conducted in which small amounts of LtEc [40] and AmEc [39] have been injected into healthy mice and rats. AmEc quickly dissociated after mixing with plasma *in vitro*, but caused no noticeable side-effects *in vivo* and all animals were healthy 18 weeks after injection. *In vitro* experiments also indicated that AmEc is not scavenged by haptoglobin, a serum protein which strongly binds to free HbA and clears it from the bloodstream.

Injection of LtEc into mice and rats also lacked any side-effects. Most importantly, no immune response was observed even after repeated injections of LtEc [40]. We have also recently transfused small amounts (0.5–1.5 g/dL) of LtEc into hamsters without any side-effects. In fact, we have observed that transfusion of LtEc causes slight vasodilation instead of the vasoconstriction caused by other HBOCs. These are only preliminary results, yet they suggest that LtEc may have a negligible rate of NO dioxygenation. LtEc also has a colloid osmotic pressure (14 mm Hg) that is higher than some HBOCs (e.g., polymerized bovine Hb, 1–5 mm Hg), but similar to human whole blood (19–24 mm Hg) [62]. Further studies with larger doses of LtEc will have to be done to accurately determine the efficacy and safety of LtEc, but all of the results presented here show that LtEc and other Ec’s are intriguing class of HBOC.

## 8. Conclusions

Altogether, these results suggest that LtEc may be a superior HBOC, since it appears to avoid many of the problems associated with other HBOC’s. It is highly stable, resistant to oxidation, and may have a negligible rate NO dioxygenation. It also has beneficial antioxidant properties which should minimize oxidative stress *in vivo*.

Despite all of the promising properties of LtEc, it is important to note that LtEc is still far away from clinical evaluation. Larger exchange transfusion studies need to be done in a series of animals (hamsters, guinea pigs, pigs, *etc*.) to determine if higher doses of LtEc pose any significant risks or side-effects. Since LtEc will be used in large doses, we must also determine the clearance mechanisms through which LtEc is eliminated *in vivo* to anticipate any toxic accumulation of heme or free iron. Models of hemorrhagic shock must also be used to simulate the effects of LtEc in emergency scenarios. *In vitro*, more work must be done to determine the exact nature of the interactions between LtEc and NO and other physiologically important ligands or effectors. The effects of H_2_O_2_ on LtEc oxidation and stability must also be determined to predict the effects of transfusing LtEc into patients with sepsis. The effects of storage conditions (pH, temperature, and buffer formulation) on LtEc must also be investigated to determine if/how LtEc can be kept for long periods of time. Once all of these questions are answered, human clinical trials with LtEc may begin.

## References

[1] Greenburg A.G., Kim H.W. (2004). Hemoglobin-based oxygen carriers. Crit. Care.

[2] Cheng D.C., Mazer C.D., Martineau R., Ralph-Edwards A., Karski J., Robblee J., Finegan B., Hall R.I., Latimer R., Vuylsteke A. (2004). A phase II dose-response study of hemoglobin raffimer (Hemolink) in elective coronary artery bypass surgery. J. Thorac. Cardiovasc. Surg..

[3] Winslow R.M. (2007). Red cell substitutes. Semin. Hematol..

[4] Caron A., Menu P., Faivre-Fiorina B., Labrude P., Alayash A.I., Vigneron C. (1999). Cardiovascular and hemorheological effects of three modified human hemoglobin solutions in hemodiluted rabbits. J. Appl. Physiol..

[5] Kasper S.M., Grune F., Walter M., Amr N., Erasmi H., Buzello W. (1998). The effects of increased doses of bovine hemoglobin on hemodynamics and oxygen transport in patients undergoing preoperative hemodilution for elective abdominal aortic surgery. Anesth. Analg..

[6] Levy J.H., Goodnough L.T., Greilich P.E., Parr G.V., Stewart R.W., Gratz I., Wahr J., Williams J., Comunale M.E., Doblar D. (2002). Polymerized bovine hemoglobin solution as a replacement for allogeneic red blood cell transfusion after cardiac surgery: Results of a randomized, double-blind trial. J. Thorac. Cardiovasc. Surg..

[7] Lamuraglia G.M., O'hara P.J., Baker W.H., Naslund T.C., Norris E.J., Li J., Vandermeersch E. (2000). The reduction of the allogenic transfusion requirement in aortic surgery with a hemoglobin-based solution. J. Vasc. Surg..

[8] Sprung J., Kindscher J.D., Wahr J.A., Levy J.H., Monk T.G., Moritz M.W., O’hara P.J. (2002). The use of bovine hemoglobin glutamer-250 (Hemopure) in surgical patients: Results of a multicenter, randomized, single-blinded tria. Anesth. Analg..

[9] Vandegriff K.D., Malavalli A., Wooldridge J., Lohman J., Winslow R.M. (2003). MP4, a new nonvasoactive PEG-Hb conjugate. Transfusion.

[10] Vandegriff K.D., Young M.A., Keipert P.E., Winslow R.M. (2007). The safety profile of Hemospan^®^: A new oxygen therapeutic designed using maleimide poly(ethylene) glycol conjugation to human hemoglobin. Transfus. Altern. Transfus. Med..

[11] Vandegriff K.D., Winslow R.M. (2009). Hemospan: Design principles for a new class of oxygen therapeutic. Artif. Organs.

[12] Vandegriff K.D., Malavalli A., Minn C., Jiang E., Lohman J., Young M.A., Samaja M., Winslow R.M. (2006). Oxidation and haem loss kinetics of poly(ethylene glycol)-conjugated haemoglobin (MP4): Dissociation between *in vitro* and *in vivo* oxidation rates. Biochem. J..

[13] Natanson C., Kern S.J., Lurie P., Banks S.M., Wolfe S.M. (2008). Cell-free hemoglobin-based blood substitutes and risk of myocardial infarction and death: A meta-analysis. J. Am. Med. Assoc..

[14] Yubisui T., Matsuki T., Tanishima K., Takeshita M., Yoneyama Y. (1977). NADPH-flavin reductase in human erythrocytes and the reduction of methemoglobin through flavin by the enzyme. Biochem. Biophys. Res. Commun..

[15] Kuma F. (1981). Properties of methemoglobin reductase and kinetic study of methemoglobin reduction. J. Biol. Chem..

[16] Scott M.D., Lubin B.H., Zuo L., Kuypers F.A. (1991). Erythrocyte defense against hydrogen peroxide: Preeminent importance of catalase. J. Lab. Clin. Med..

[17] Liu X., Miller M.J., Joshi M.S., Sadowska-Krowicka H., Clark D.A., Lancaster J.R. (1998). Diffusion-limited reaction of free nitric oxide with erythrocytes. J. Biol. Chem..

[18] Bunn H.F., Briehl R.W. (1970). The interaction of 2,3-diphosphoglycerate with various human hemoglobins. J. Clin. Invest..

[19] Chiancone E. (1968). Dissociation of hemoglobin into subunits. II. Human oxyhemoglobin: Gel filtration studies. J. Biol. Chem..

[20] Terwilliger R.C. (1980). Structures of invertebrate hemoglobins. Am. Zool..

[21] Boffi A., Verzili D., Chiancone E., Leone M., Cupane A., Militello V., Vitrano E., Cordone L., Yu W., di Iorio E.E. (1994). Stereodynamic properties of the cooperative homodimeric Scapharca inaequivalvis hemoglobin studied through optical absorption spectroscopy and ligand rebinding kinetics. Biophys. J..

[22] Di Iorio E., Tavernelli I., Yu W. (1997). Dynamic properties of monomeric insect erythrocruorin III from Chironomus thummi-thummi: Relationships between structural flexibility and functional complexity. Biophys. J..

[23] Zal F., Lallier F.H., Wall J.S., Vinogradov S.N., Toulmond A. (1996). The multi-hemoglobin system of the hydrothermal vent tube worm Riftia pachyptila. I. Reexamination of the number and masses of its constituents. J. Biol. Chem..

[24] Royer W.E., Sharma H., Strand K., Knapp J.E., Bhyravbhatla B. (2006). Lumbricus erythrocruorin at 3.5 A resolution: Architecture of a megadalton respiratory complex. Structure.

[25] Royer W.E., Omartian M.N., Knapp J.E. (2007). Low resolution crystal structure of Arenicola erythrocruorin: Influence of coiled coils on the architecture of a megadalton respiratory protein. J. Mol. Biol..

[26] Terwilliger N., Terwilliger R.C. (1978). Oxygen binding domains of a clam (Cardita borealis) extracellular hemoglobin. Biochim. Biophys. Acta.

[27] Xu Y., Zheng Y., Fan J.-S., Yang D. (2006). A new strategy for structure determination of large proteins in solution without deuteration. Nat. Methods.

[28] Royer W.E. J., Strand K., van Heel M., Hendrickson W.A. (2000). Structural hierarchy in erythrocruorin, the giant respiratory assemblage of annelids. Proc. Natl. Acad. Sci. USA.

[29] Strand K., Knapp J.E., Bhyravbhatla B., Royer W.E. (2004). Crystal structure of the hemoglobin dodecamer from Lumbricus erythrocruorin: Allosteric core of giant annelid respiratory complexes. J. Mol. Biol..

[30] Fushitani K., Matsuura M.S., Riggs A.F. (1988). The amino acid sequences of chains a, b, and c that form the trimer subunit of the extracellular hemoglobin from Lumbricus terrestris. J. Biol. Chem..

[31] Xie Q., Donahue R.A., Schneider K., Mirza U.A., Haller I., Chait B.T., Riggs A.F. (1997). Structure of chain d of the gigantic hemoglobin of the earthworm. Biochim. Biophys. Acta.

[32] Suzuki T., Riggs A.F. (1993). Linker chain L1 of earthworm hemoglobin. Structure of gene and protein: Homology with low density lipoprotein receptor. J. Biol. Chem..

[33] Kao W.Y., Qin J., Fushitani K., Smith S.S., Gorr T.A., Riggs C.K., Knapp J.E., Chait B.T., Riggs A.F. (2006). Linker chains of the gigantic hemoglobin of the earthworm Lumbricus terrestris: Primary structures of linkers L2, L3, and L4 and analysis of the connectivity of the disulfide bonds in linker L1. Proteins.

[34] Sharma P.K., Kuchumov A.R., Chottard G., Martin P.D., Wall J.S., Vinogradov S.N. (1996). The role of the dodecamer subunit in the dissociation and reassembly of the hexagonal bilayer structure of Lumbricus terrestris hemoglobin. J. Biol. Chem..

[35] Lamy M.L., Daily E.K., Brichant J.F., Larbuisson R.P., Demeyere R.H., Vandermeersch E.A., Lehot J.J., Parsloe M.R., Berridge J.C., Sinclair C.J. (2000). Randomized trial of diaspirin cross-linked hemoglobin solution as an alternative to blood transfusion after cardiac surgery. The DCLHb cardiac surgery trial collaborative group. Anesthesiology.

[36] Numoto N., Nakagawa T., Kita A., Sasayama Y., Fukumori Y., Miki K. (2005). Structure of an extracellular giant hemoglobin of the gutless beard worm Oligobrachia mashikoi. Proc. Natl. Acad.Sci. USA.

[37] Lamy J., Kuchumov A.R., Taveau J.C., Vinogradov S.N., Lamy J.N. (2000). Reassembly of Lumbricus terrestris hemoglobin: A study by matrix-assisted laser desorption/ionization mass spectrometry and 3D reconstruction from frozen-hydrated specimens. J. Mol. Biol..

[38] Birukou I., Soman J., Olson J.S. (2011). Blocking the gate to ligand entry in human hemoglobin. J. Biol. Chem..

[39] Rousselot M., Delpy E., Drieu La Rochelle C., Lagente V., Pirow R., Rees J.F., Hagege A., Le Guen D., Hourdez S., Zal F. (2006). Arenicola marina extracellular hemoglobin: A new promising blood substitute. Biotechnol. J..

[40] Hirsch R.E., Jelicks L.A., Wittenberg B.A., Kaul D.K., Shear H.L., Harrington J.P. (1997). A first evaluation of the natural high molecular weight polymeric Lumbricus terrestris hemoglobin as an oxygen carrier. Artif. Cells Blood Substit. Immobil. Biotechnol..

[41] Zapletal C., Bode A., Lorenz M.W., Gebhard M.M., Golling M. (2009). Effects of hemodilution with a hemoglobin-based oxygen carrier (HBOC-201) on ischemia/reperfusion injury in a model of partial warm liver ischemia of the rat. Microvasc. Res..

[42] Standley P., Mainwaring M.G., Gotoh T., Vinogradov S.N. (1988). The calcium, copper and zinc content of some annelid extracellular haemoglobins. Biochem. J..

[43] Harrington J.P. (1994). Multimeric Lumbricus hemoglobin stabilization by alkali and alkaline earth cations. Comp. Biochem. Physiol. Part A.

[44] Chiancone E., Vecchini P., Rossi Fanelli M.R., Antonini E. (1972). Studies on erythrocruorin. II. Dissociation of earthworm erythrocruorin. J. Mol. Biol..

[45] Smith M.L., Paul J., Ohlsson P.I., Paul K.G. (1997). The spontaneous hemin release from Lumbricus terrestris hemoglobin. Comp. Biochem. Physiol. Part A.

[46] Fushitani K., Imai K., Riggs A.F. (1986). Oxygenation properties of hemoglobin from the earthworm, Lumbricus terrestris. Effects of pH, salts, and temperature. J. Biol. Chem..

[47] Ochiai T., Weber R.E. (2002). Effects of magnesium and calcium on the oxygenation reaction of erythrocruorin from the marine polychaete Arenicola marina and the terrestrial oligochaete Lumbricus terrestris. Zool. Sci..

[48] Vidugiris G., Harrington J.P., Friedman J.M., Hirsch R.E. (1993). Absence of ligand binding-induced tertiary changes in the multimeric earthworm Lumbricus terrestris hemoglobin. A resonance Raman study. J. Biol. Chem..

[49] Fushitani K., Riggs A.F. (1991). The extracellular hemoglobin of the earthworm, Lumbricus terrestris. Oxygenation properties of isolated chains, trimer, and a reassociated product. J. Biol. Chem..

[50] Alayash A.I. (2000). Hemoglobin-based blood substitutes and the hazards of blood radicals. Free Radic. Res..

[51] Stellwagen E. (1978). Haem exposure as the determinate of oxidation-reduction potential of haem proteins. Nature.

[52] Gow A.J., Payson A.P., Bonaventura J. (2005). Invertebrate hemoglobins and nitric oxide: How heme pocket structure controls reactivity. J. Inorg. Biochem..

[53] Harrington J.P., Kobayashi S., Dorman S.C., Zito S.L., Hirsch R.E. (2007). Acellular invertebrate hemoglobins as model therapeutic oxygen carriers: Unique redox potentials. Artif. Cells Blood Substit. Immobil. Biotechnol..

[54] Dorman S.C., Kenny C.F., Miller L., Hirsch R.E., Harrington J.P. (2002). Role of redox potential of hemoglobin-based oxygen carriers on methemoglobin reduction by plasma components. Artif. Cells Blood Substit. Immobil. Biotechnol..

[55] Dorman S.C., Harrington J.P., Martin M.S., Johnson T.V. (2004). Determination of the formal reduction potential of Lumbricus terrestris hemoglobin using thin layer spectroelectrochemistry. J. Inorg. Biochem..

[56] Harnois T., Rousselot M., Rogniaux H., Zal F. (2009). High-level production of recombinant Arenicola marina globin chains in Escherichia coli: A new generation of blood substitute. Artif. Cells Blood Substit. Immobil. Biotechnol..

[57] Liochev S.I., Kuchumov A.R., Vinogradov S.N., Fridovich I. (1996). Superoxide dismutase activity in the giant hemoglobin of the earthworm, Lumbricus terrestris. Arch. Biochem. Biophys..

[58] Giacometti G.M., Focesi A., Brunori M., Wyman J. (1975). Effect of light on carbon monoxide binding by erythrocruorin. J. Biol. Chem..

[59] Giacometti G.M., Focesi A., Giardina B., Brunori M., Wyman J. (1975). Kinetics of binding of carbon monoxide to lumbricus erythrocruorin: A possible model. Proc. Natl. Acad. Sci. USA.

[60] Eich R., Li T., Lemon D.D., Doherty D.H., Curry S., Aitken J.F., Johnson K.A., Smith R.D., Phillips G.N., Olson J.S. (1996). Mechanism of NO-induced oxidation of myoglobin and hemoglobin. Biochemistry.

[61] Olson J.S., Eich R.F., Smith L.P., Warren J.J., Knowles B.C. (1997). Protein engineering strategies for designing more stable hemoglobin-based blood substitutes. Artif. Cells Blood Substit. Immobil. Biotechnol..

[62] Elmer J., Zorc K., Rameez S., Zhou Y., Cabrales P., Palmer A.F. (2012). Hypervolemic infusion of *Lumbricus terrestris* erythrocruorin purified by tangential flow filtration. Transfusion.

[63] Santiago P.S., Carvalho J.W., Domingues M.M., Santos N.C., Tabak M. (2010). Thermal stability of extracellular hemoglobin of Glossoscolex paulistus: Determination of activation parameters by optical spectroscopic and differential scanning calorimetric studies. Biophys. Chem..

[64] Hourdez S., Weber R.E. (2005). Molecular and functional adaptations in deep-sea hemoglobins. J. Inorg. Biochem..

[65] Bachega J.F.R., Bleicher L., Horjales E.R., Santiago P.S., Garratt R.C., Tabak M. (2010). Crystallization and preliminary structural analysis of the giant haemoglobin from *Glossoscolex paulistus* at 3.2 Å. J. Synchrotron Radiat..

[66] Poli A.L., Moreira L.M., Hidalgo A.A., Imasato H. (2005). Autoxidation studies of extracellular hemoglobin of *Glossoscolex paulistus* at pH 9: Cyanide and hydroxyl effect. Biophys. Chem..

[67] Poli A.L., Moreira L.M., Imasato H. (2011). Autoxidation of giant extracellular hemoglobin of *Glossoscolex paulistus*: Molecular mechanism and oligomeric implications. Spectrochim. Acta A.

[68] Agustinho S.C., Tinto M.H., Imasato H., Tominaga T.T., Perussi J.R., Tabak M. (1298). Spectroscopic studies of the met form of the extracellular hemoglobin from *Glossoscolex paulistus*. Biochim. Biophys. Acta.

[69] Bispo J.A., Santos J.L., Landini G.F., Goncalves J.M., Bonafe C.F. (2007). pH dependence of the dissociation of multimeric hemoglobin probed by high hydrostatic pressure. Biophys. Chem..

[70] Moreira L.M., Poli A.L., Lyon J.P., Saade J., Costa-Filho A.J., Imasato H. (2008). Ferric species of the giant extracellular hemoglobin of *Glossoscolex paulistus* as function of pH: An EPR study on the irreversibility of the heme transitions. Comp. Biochem. Physiol. B.

[71] Cardillo F., de Paula E., Oliveira G.R., Marangoni S., Oliviera B., Meirelles N.C. (1997). Erythrocruorin of *Glossoscolex paulistus* (Oligochaeta, Glossoscolecidae): Modulation of oxygen affinity by specific antibodies. Biochem. Mol. Biol. Int..

[72] Bonafe C.F., Villas-Boas M., Suarez M.C., Silva J.L. (1991). Reassembly of a large multisubunit protein promoted by nonprotein factors. Effects of calcium and glycerol on the association of extracellular hemoglobin. J. Biol. Chem..

[73] Shander A., Hofmann A., Gombotz H., Theusinger O.M., Spahn D.R. (2007). Estimating the cost of blood: Past, present, and future directions. Best Pract. Res. Clin. Anaesthesiol..

